# Recent Advances in Chemoenzymatic Peptide Syntheses

**DOI:** 10.3390/molecules190913755

**Published:** 2014-09-03

**Authors:** Kenjiro Yazawa, Keiji Numata

**Affiliations:** Enzyme Research Team, Biomass Engineering Program Cooperation Division, RIKEN, Center for Sustainable Resource Science, 2-1 Hirosawa, Wako-shi, Saitama 351-0198, Japan

**Keywords:** chemoenzymatic, peptide, protease, enzyme, reaction mechanisms, reaction media, substrate mimetics, enzyme engineering

## Abstract

Chemoenzymatic peptide synthesis is the hydrolase-catalyzed stereoselective formation of peptide bonds. It is a clean and mild procedure, unlike conventional chemical synthesis, which involves complicated and laborious protection-deprotection procedures and harsh reaction conditions. The chemoenzymatic approach has been utilized for several decades because determining the optimal conditions for conventional synthesis is often time-consuming. The synthesis of poly- and oligopeptides comprising various amino acids longer than a dipeptide continues to pose a challenge owing to the lack of knowledge about enzymatic mechanisms and owing to difficulty in optimizing the pH, temperature, and other reaction conditions. These drawbacks limit the applications of the chemoenzymatic approach. Recently, a variety of enzymes and substrates produced using recombinant techniques, substrate mimetics, and optimal reaction conditions (e.g., frozen aqueous media and ionic liquids) have broadened the scope of chemoenzymatic peptide syntheses. In this review, we highlight the recent advances in the chemoenzymatic syntheses of various peptides and their use in developing new materials and biomedical applications.

## 1. Introduction

Linear and circular peptides play important roles in biological reactions and are therefore considered important target molecules for pharmaceutical, nutritional, and cosmetic applications [1]. Peptides are formed when the amino group of one amino acid forms an amide (peptide) linkage with the carboxyl group of another amino acid. Various methods for synthesizing peptides have been developed. These include solid phase peptide synthesis (SPPS) and recombinant bacterial expression systems [2,3]. However, these methods have several drawbacks. Although the amino acid sequence can be precisely controlled during SPPS, a laborious protection-deprotection regimen, along with harsh reaction conditions and the use of toxic reagents makes this procedure difficult [4]. In the recombinant expression system, post-translational modification can be performed; however, the yields are often low and purification procedures are time-consuming [4]. Although proteases can be easily utilized for hydrolyzing proteins, they also function in the reverse direction, thereby facilitating peptide synthesis. This phenomenon has been known since the middle of the 20th century [5,6]. It is still difficult to synthesize natural polypeptides by a chemoenzymatic approach, however, chemoenzymatic synthesis offers a flexible and more specific method for peptide synthesis [7]. Enzymatic reactions usually occur under mild conditions [8]. More importantly, enzymatic chemoselectivity allows the conversion of non-side chain protected peptides and the absolute absence of racemization which is a considerable limitation of existing peptide coupling agents [9]. Chemoenzymatic synthesis strongly supports green chemistry [4]. This review highlights the recent advances in chemoenzymatic peptide synthesis. We have discussed the corresponding reaction mechanisms and listed the frequently used enzymes. In addition, we have described the engineered reaction conditions as well as the properties of peptides for the development of new materials and biomedical applications.

## 2. Reaction Mechanisms

Enzymes function optimally in their native environment. Their unique catalytic abilities sometimes depend on the pH, substrate concentration, and the presence of organic solvents [10]. Enzymatic catalysis can be regulated by the characteristics of the enzymes. Generally, proteases are known for their hydrolysis efficiency, but they can also function in the opposite direction, leading to the formation of peptide bonds (aminolysis) [1,10]. Hydrolysis and aminolysis often compete with each other. However, enzymes prefer aminolysis to hydrolysis under certain specific conditions, such as the presence of large amounts of the substrate, despite being surrounded by excess water.

**Figure 1:**
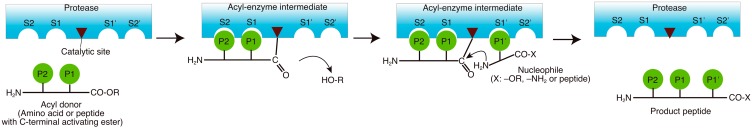
Protease-catalyzed peptide synthesis according to the nomenclature by Schechter and Berger [11].

According to the nomenclature by Schechter and Berger, acyl donors need to be recognized in the S subsite of the enzyme. In addition, nucleophiles are recognized in the S' region (Figure 1) [11]. The reaction rate is determined mainly by the specificity of the enzyme toward the acyl donor, and specific binding of the nucleophile to the S' subsite of the protease is critical for ensuring high yields [12].

**Scheme 1:**
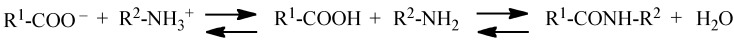
Equilibrium-controlled chemoenzymatic synthesis of peptide.

Protease-catalyzed peptide synthesis is either equilibrium-controlled or kinetically controlled [4,8]. In equilibrium-controlled synthesis, all the proteases are usually consumed and a free carboxyl group acts as an acyl donor [4,8]. Proteases improve the reaction rate, but do not affect the final equilibrium of the reaction. Proton transfer occurs during the first ionization step, followed by condensation, as shown in Scheme 1 [4]. The major drawbacks of the equilibrium-controlled synthesis include low yield and a slow reaction rate as compared to the kinetically controlled reactions. Larger quantities of the enzyme are also needed [1]. Optimal reaction conditions are required for directing the reaction toward peptide synthesis. An organic solvent shifts the equilibrium toward peptide synthesis when enzymatic products are soluble in it. In addition, organic solvents are also useful for preventing the hydrolysis of the protease-acyl complex, owing to the reduced quantities of water included in the reaction. However, organic solvents can also adversely affect the enzymes [13].

**Scheme 2:**
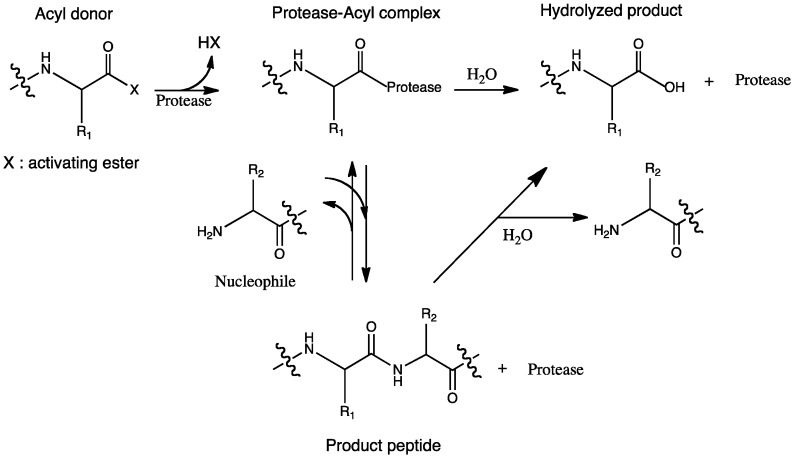
Kinetically-controlled chemoenzymatic synthesis of peptide.

During kinetically controlled synthesis, an activated ester is needed for donating the acyl group. Preparation of suitable acyl donors without racemization is a considerable challenge, however, ruthenium-catalyzed synthesis of peptide enol esters is a good example of a way to solve this problem [9]. Serine and cysteine proteases characteristically form reactive acyl enzyme intermediates during catalysis and are the preferred candidates for the reaction. They play an important role in transferring the acyl species to the amino groups of amino acids or peptides [14]. The acyl-enzyme complex is transported in two directions: aminolysis involves the nucleophilic attack of the amine in the substrate, whereas hydrolysis involves the attack by the water molecule, as shown in Scheme 2.

The reaction substrate decreases gradually during the course of the reaction, and the effect of hydrolysis increases. It is therefore important to stop the aminolysis reaction before the rate of hydrolysis exceeds the rate of aminolysis [1]. The success of the aminolysis reaction depends on the aminolysis:hydrolysis ratio, which is determined by many factors such as enzyme specificity and substrate concentration, as well as the presence of activated carboxyl groups.

## 3. Enzymes Used in the Peptide Synthesis

In chemoenzymatic peptide synthesis, cysteine protease (papain, bromelain), serine protease (α-chymotrypsin, proteinase K, trypsin, subtilisin), and esterase (lipase) are used in many cases, as shown in Table 1. Engineered proteases are also created by random mutagenesis, site-directed mutagenesis, and encapsulation in silica nanospheres to enhance its activity, stability, and selectivity.

**Table 1:** Chemoenzymatic peptide syntheses.

Protease	Monomer	pH	Temp.	Substrate	Enzyme	Yield	DP_avg_	Ref
papain	Ala-OEt	11	40 °C	0.7 M	7.1 mg/mL	36%	13.5 (NMR)	[15]
papain	Ala-OEt	7	40 °C	0.7 M	7.1 mg/mL	67%	9.3 (NMR)	[15]
papain	Tyr-OEt	7	25 °C	0.2 M	20 mg/mL	64%	12 (SEC)	[16]
papain	Glu-(OEt)_2_	7	40 °C	0.5 M	40 mg/mL	60%	9.5 (NMR)	[17]
papain	Glu-(OEt)_2_Cys-OEt	8	40 °C	0.2 M,0.3 M	16 units/mL	34%	9.2 (NMR)	[18]
papain	Tyr-OEt	7	40 °C	0.2 M	12 mg/mL	80%	9 (NMR)	[19]
papain	Ala-Gly-OEt	9	40 °C	0.5 M	20 mg/mL	48%	9.5 (NMR)	[20]
papain	Leu-OEt	7	40 °C	1.0 M	10 mg/mL	25%	6–9 (MS)	[21]
papain	Tyr-OEtLys-OEt	9.5	40 °C	0.3 M0.3 M	7.0 mg/mL	42%	8 (NMR)	[22]
bromelain	Phe-OEt	8	40 °C	0.1 M	18.6 mg/mL	45%	8.2 (NMR)	[23]
bromelain	Lys-OEt	7.6	40 °C	0.5 M	20 mg/mL	80%	3.6 (NMR)	[24]
α-chymotrypsin	Cys-OEt	8	−20 °C	0.1 M	20 μM	85%	6–11 (MS)	[25]
α-chymotrypsin	Lys-Leu-OEt	8.5	40 °C	0.3 M	10 mg/mL	-	4.7 (NMR)	[26]
proteinase K	Phe-OEt	8	40 °C	0.6 M	1.0 mg/mL	65%	12 (NMR)	[27]
trypsin	Lys-OEt	10	25 °C	0.2 M	10 μM	50%	2–8 (MS)	[28]
trypsin	Arg-OEt	10	25 °C	0.5 M	10 μM	43%	-	[29]
subtilisin	Bz-Arg-OEtGly-NH_2_	10	45 °C	0.05 M0.4 M	-	83%	-	[30]
lipase	Bz-Arg-OEtGly-Asp-Ser-NH_2_	7.5	10 °C	0.05 M0.4 M	10 mg/mL	74%	-	[31]
lipase	2-azetidinone	-	90 °C	0.3 M	20 mg/mL	73%	8 (NMR)	[32]

### 3.1. Cysteine Protease

#### 3.1.1. Papain

Papain is the English translation of “papaïne” assigned by Wurtz and Bouchut to the proteolytically active constituent in the latex of the tropical papaya fruit (*Carica papaya*) [33]. Papain exhibits endopeptidase, amidase, and esterase activities [33]. Papain shows broad substrate specificity. The hydrophobic pocket in the S2 subsite is essential for substrate incorporation and recognition of the bulky aromatic residues [34]. The S1 subsite is not as selective as the S2 subsite. Although there is some preference for Arg and Lys at this position, the Val in the S1 region prevents substrate recognition [33,35]. Papain is stable and active under a wide range of conditions from pH 4 to pH 10 and at temperatures up to 80 °C [33].

Papain-catalyzed synthesis is performed using l-alanine ethyl ester as the monomeric substrate for poly(l-alanine). Reactions carried out at an alkaline pH cause a higher degree of polymerization (DP) than those carried out at a neutral pH [15]. Papain is also used for synthesizing poly(l-tyrosine) [16] and poly(l-glutamic acid) [17] using l-tyrosine ethyl ester hydrochloride and l-glutamic acid diethyl ester hydrochloride as the substrates, respectively. The protease-catalyzed copolymerization of amino acids is first achieved by papain using l-glutamic acid ester and various amino acid esters as substrates [17]. Interestingly, l-aspartic acid diethyl ester and alanine ethyl ester are not subjected to the papain-catalyzed homopolymerization. However, they are copolymerized in the presence of l-glutamic acid and papain [17]. Co-oligomerization of l-Et_2_-Glu and l-Et-Cys at different feed ratios is successfully catalyzed by papain [18]. By using an l-Et_2_-Glu:l-Et-Cys feed ratio of 4:6, the co-oligomer is obtained with 47 mol% l-Cys residues and with an average DP (DP_avg_) of 9 [18]. Papain-catalyzed oligomerization of four hydrophobic amino acids (Leu, Tyr, Phe, and Trp) has been precisely investigated for determining the preferences in substrate recognition for homo-oligomerization, binary co-oligomerization, and ternary co-oligomerization [19]. Diblock and random co-oligopeptides of l-lysine and l-alanine are successfully synthesized by papain [36]. Alternating oligopeptides are synthesized by papain-mediated catalysis using the dipeptide monomer, Ala-Gly ethyl ester, which results in an 80% yield at pH 7.5 with DP_avg_ = 9.4 [20]. Papain-catalyzed oligomerization of l-lysine protected at the N_ε_-position with the *tert*-butoxycarbonyl or carboxybenzyl group ensures a higher yield and a simpler purification process owing to the precipitation-driven reaction derived from the hydrophobicity of the protected group [37].

#### 3.1.2. Bromelain

Bromelain is abundant in the stem of the pineapple plant and shows a broad specificity for the cleavage of proteins; preferences for polar amino acids in both the P1 as well as the P1' positions have been reported [38]. The optimal pH range for protein substrates is broad. However, most assays are performed near the neutral pH [38]. The oligomerization of the l-phenylalanine ethyl ester is catalyzed by bromelain in the presence of water-miscible organic cosolvents, which leads to better yields [23]. Oligo(l-lysine) from the l-lysine ethyl ester is synthesized using four proteases (papain, bromelain, α-chymotrypsin, and trypsin) [24]. Bromelain is preferred over the other proteases because it gives the highest yield and average chain length [24]. A percent monomer conversion of 84% ± 2% and a DP_avg_ of 4.1 have been obtained in an earlier study by maintaining the pH between 7.6 and 7.8 [24].

### 3.2. Serine Protease

#### 3.2.1. α-Chymotrypsin

Chymotrypsin has been identified as the major protease component of pancreatic juice [39]. The major cleavage sites of α-chymotrypsin involve peptide bonds, wherein the carboxyl side is a hydrophobic amino acid such as tyrosine, tryptophan, and phenylalanine [39]. These amino acids contain an aromatic ring in the side chain that fits into a hydrophobic region of the enzyme. The hydrophobicity and the shape complementarity between the peptide substrate P1 side chain and the enzyme S1 binding cavity account for the substrate specificity of this enzyme [34,39]. The enzyme α-chymotrypsin also hydrolyzes other amide bonds in the peptide, particularly those containing leucine and methionine at the P1 position, at slower rates [39].

Poly-l-cysteine with the DP ranging between 6 and 11 is synthesized via α-chymotrypsin catalysis in a frozen aqueous medium without blocking the SH groups [25]. This enzyme-catalyzed oligomerization of the alternating peptides of lysine and leucine using lysine-leucine-ethyl ester is performed in aqueous media at pH 8.5 [26]. Papain causes loss of sequence control, probably owing to the competitive transamidation and hydrolysis reactions [26]. α–Chymotrypsin-catalyzed formation of His-Lys bonds can be carried out both in the aqueous as well as the frozen aqueous solutions. Freezing tends to increase the corresponding reaction yield [40].

#### 3.2.2. Proteinase K

The alkaline protease secreted into the culture medium by the mold *Tritirachium album* Limber is commonly known as proteinase K [41]. The designation “K” was chosen to indicate that it can even hydrolyze native keratin. Proteinase K shows broad substrate specificity and cleaves the peptide bond next to the carboxyl group of aliphatic and aromatic amino acids [42,43]. In addition, proteinase K also hydrolyzes esters [41,43]. Proteinase K is active in a broad pH range, that is, from pH 7.5 to pH 12.0 [42], and remains active even at temperatures as high as 60 °C [41].

Proteinase K-catalyzed peptide synthesis was first detected in linear oligo(l-phenylalanine) and in the star oligopeptides on using tris(2-aminoethyl)amine as the branching terminator [27]. With regard to the ability for processing the substrate monomer, proteinase K is approximately 30 times more effective than bromelain during the synthesis of oligo(l-phenylalanine) [27].

#### 3.2.3. Trypsin

Trypsin was first detected and identified in 1876 by Kühne owing to its proteolytic activity in pancreatic secretions [44]. Trypsin shows specificity toward the peptide bonds at the C-terminal side of lysine and arginine residues, except for the -Arg-Pro- and -Lys-Pro- bonds [45]. A pH of 8 is optimal for the activity of trypsin [44]. The trypsin-catalyzed oligomerization of the l-lysine ethyl ester shows an overall reaction yield of 70% after 2 h of the reaction, at a pH of 10 [28]. Trypsin is also used to synthesize peptides containing histidine [40] and arginine [29].

#### 3.2.4. Subtilisin (Alcalase)

Subtilisin, also known as alcalase, is an extracellular serine endoprotease and the name derives from the bacterial species, *Bacillus subtilis*, from which the enzyme was isolated [46]. Subtilisins show good stability in the pH range 7–10 [46]. Synthesis of dipeptide Bz-Arg-Gly-NH_2_ was catalyzed by subtilisin in water/organic cosolvent systems, which resulted in 83% yield [30]. Subtilisin-catalyzed fragment condensation strategy was established in neat organic solvent using larger peptide fragments up to the 10-mer level bearing multiple side chain-protecting groups [47].

### 3.3. Lipase

Lipases catalyze the hydrolysis of triglycerides into the corresponding fatty acids and glycerol [48]. In addition, lipases catalyze many other reactions such as esterification, amidation, and transesterification. Moreover, lipases accept a wide variety of substrates while maintaining their regioselectivity and stereoselectivity. Lipases are highly stable even under adverse conditions such as organic solvents and high temperatures [48]. Lipases are used to catalyze the formation of the tetrapeptide Benzoyl-Arg-Gly-Asp-Ser-NH_2_ in aqueous water-miscible organic cosolvent systems with benzoyl-Arg ethyl ester as the acyl donor and the tripeptide Gly-Asp-Ser-NH_2_ as the nucleophile [31]. Polyamide (poly-β-alanine) is synthesized by *Candida antarctica* lipase B immobilized on polyacrylic resin (Novozyme 435), which catalyzes the ring-opening of the cyclic starting material 2-azetidinone [32].

### 3.4. Engineered Proteases

The stability of α-chymotrypsin in which Met192 is modified into methionine sulfoxide remains at 80% for >4 h at a pH of 9 [49]. The nucleophilicity of the amine group is higher at an alkaline pH than at neutral pH, and α-chymotrypsin methylated at the ε_2_-N of the active site histidine also shows efficient catalysis in peptide synthesis [49]. Site-directed mutagenesis has been utilized to design trypsin variants with decreased rates of proteolytic side reactions [50]. Starting from the trypsin variant D189S which is already known for its low amidase activity, both Ser189 and Ser190 were mutated into Ala to further repress the inherent amidase activity of the mutant trypsin D189S [50]. Doubly mutated subtilisin (Q103R and N218S) showed 10-fold higher activity than the wild type in 20% dimethylformamide (DMF) and was twice as stable as the wild type in 40% DMF [51]. The encapsulation of three different proteases (papain, bromelain, and trypsin) in silica nanospheres was performed using sodium metasilicate as a silica precursor and ethyleneamines as initiators, which resulted in a 10 °C increase in the optimal temperature during the production of poly-l-leucine [21].

## 4. Design of Reaction Conditions

Optimization of pH, temperature, and concentrations of the substrate and enzymes are essential to ensure better yield and a higher DP in the chemoenzymatic peptide synthesis. The novel reaction media (including frozen solution, ionic liquid, and supercritical carbon dioxide) used recently increased the efficiency of the chemoenzymatic peptide synthesis. Engineered substrates and enzymes thus broaden the advantage of the chemoenzymatic approach.

### 4.1. pH, Temperature, and Substrate and Enzyme Concentrations

During chemoenzymatic peptide synthesis, the pH, temperature, and substrate and enzyme concentrations dramatically influence the DP as well as the yield of the reaction. The pH range at which the protease and substrate are active and reactive should always be determined before initiating the procedure. Alkaline conditions are generally suitable for the aminolysis reaction of peptides owing to the p*K*_a_ of the amine groups of the monomeric substrates. The deprotonated amino group is preferred for the initial nucleophilic attack on the protease-acyl complex by the incoming nucleophile (Scheme 2) [23,24,27,37]. A relatively high pH often leads to the hydrolysis of the ester substrate during the kinetically controlled synthesis.

The optimal temperature for the protease-catalyzed peptide synthesis depends on the enzyme and the substrate. To date, the aminolysis reactions have usually been performed around 40 °C, thereby giving higher yields [23,24,27]. This limited temperature range is due to the lack of variety of enzyme and monomeric substrates. To investigate a wider temperature range, engineered enzymes, unnatural amino acids, and artificial monomers need to be used in aminolysis reactions.

The substrate concentration also affects the reaction of the acyl-enzyme complex, resulting in aminolysis or hydrolysis. Excess concentration of the substrate is needed for aminolysis to proceed to peptide synthesis. The concentrations of the monomeric substrates ranged from 0.1 to 1.0 M in some recent studies [23,24,27]. Moreover, the concentration of the enzyme ranged from 0.5 to 40 mg/mL during the aminolysis reaction. This led to efficient reaction catalysis and also prevented the hydrolysis of the substrate itself, owing to a shorter reaction time needed for the peptide synthesis [23,24,27,37].

### 4.2. Frozen Aqueous Media

It has been reported that frozen aqueous solutions prevent the hydrolysis of the substrate and the synthesized peptide, while not affecting the ability to catalyze aminolysis. An α-chymotrypsin-catalyzed peptide synthesis was conducted using N-benzyloxycarbonyl-phenylalanine carbamoylmethyl ester and H-Phe-NH_2_ as the acyl donor and nucleophile, respectively, in a frozen aqueous solution (−24 °C). The yield increased up to 90% [52,53]. Poly-l-cysteine was synthesized in a frozen aqueous solution at −20 °C using l-cysteine ethyl ester (Cys-OEt) as a substrate and α-chymotrypsin as the catalytic enzyme, which provided poly-l-cysteine with a DP ranging from 6 to 11 and also provided a yield of 85% [25].

### 4.3. Ionic Liquids

**Figure 2:**
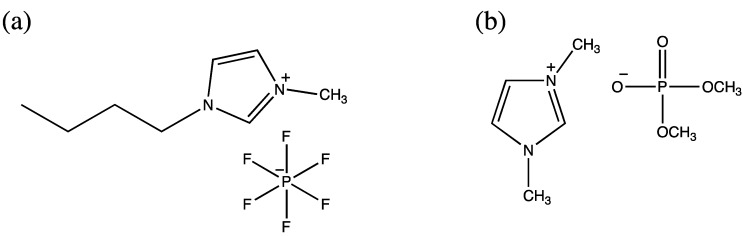
(**a**)1-Butyl-3-methylimidazolium hexafluorophosphate; (**b**)1,3-Dimethyl-imidazolium dimethylphosphate.

Ionic liquids constitute a relatively newer class of nearly nonvolatile, purely ionic materials offering huge potential for the optimization of reaction processes owing to their adaptable, diverse, and unique set of physicochemical properties that lead evidently to remarkable improvements as compared to conventional solvent systems [54,55]. The first enzymatic peptide synthesis in ionic liquid was reported by Erbeldinger *et al.* in 2000 [56]. Carboxybenzyl-aspartame was synthesized using thermolysin as a catalyst and carboxybenzyl-l-aspartate and l-phenylalanine methyl ester hydrochloride as substrates in 1-butyl-3-methylimidazolium hexafluorophosphate (Figure 2a) containing 5% (v/v) water, which resulted in a 95% yield [56]. The truncated sequence (46–92) of the peptidyl prolyl cis/trans isomerase parvulin 10 from *Escherichia coli* was selected as the synthesis target, and α-chymotrypsin-catalyzed formation of the peptide bond was successfully conducted between the 4-guanidinophenyl ester of the peptide (46–62) and another peptide (63–92) in a reaction medium comprising 60% (v/v) 1,3-dimethylimidazolium dimethylphosphate (Figure 2b) and 40% MOPS buffer, pH 8.0. This resulted in a 52% yield [54].

### 4.4. Supercritical Carbon Dioxide

Carbon dioxide is a supercritical fluid utilized as an environmentally benign solvent for enzymatic reactions [57]. α-Chymotrypsin-catalyzed dimer synthesis from *N*-acetyl-l-tyrosine ethyl ester and glycine amides was performed in 20% (v/v) supercritical carbon dioxide in an acetonitrile/supercritical carbon dioxide system, which resulted in a 91% yield for a 5 h reaction [58]. Surfactant-coated α-chymotrypsin complexes were used to synthesize dipeptides from *N*-acetyl-Phe-OEt and Gly-NH_2_ in supercritical carbon dioxide at 308.2 K [57]. The nonionic surfactant, sorbitan monostearate, was used to prepare a complex with α-chymotrypsin, which resulted in 96% conversion after a 12 h reaction in supercritical carbon dioxide at 10.1 MPa.

### 4.5. Immobilization of Substrate or Enzyme

There is considerable interest in using an immobilized substrate on a solid support such as poly(ethylene glycol)-acrylamide, which has been shown to be accessible to small enzymes [59]. Thermolysin-catalyzed synthesis of a dimer (Phe-Phe) was achieved with 99% yield, using Fmoc-phenylalanine and phenylalanine immobilized on poly(ethylene glycol)-acrylamide in phosphate buffer, pH 7.5 [59]. Suppressed ionization of the amine on the solid support due to the overall positive charge of the resin may be contributed to high yield. In addition, improved solvation of the hydrophobic acyl donors in the poly(ethylene glycol)-acrylamide resin may result in a higher local concentration of the acyl donor near the site of catalysis [59].

Immobilized enzymes have numerous advantages. For instance, they show better stability than the free enzymes in many cases. Moreover, the enzyme can be used repeatedly [60]. Papain immobilized on epoxy particles and α-chymotrypsin immobilized on Eupergit C have been utilized for the stepwise synthesis of cholecystokinin-5, which is a pentapeptide comprising Gly-Trp-Met-Asp-Phe [61]. Cholecystokinin-5 is a key intermediate during the synthesis of cholecystokinin-8, which plays a physiological role as a neurotransmitter working in the central nervous system.

### 4.6. Cross-Linked Enzyme Aggregate

The formation of cross-linked enzyme aggregates (CLEAs), which involve cross-linking with a bifunctional reagent and enzymes, has emerged as a novel and versatile carrier-free immobilization technique [62,63]. The ester conversion and subsequent dipeptide formation were performed in the presence of alcalase-CLEA using a one-pot approach [64]. Starting from an N-terminal carboxybenzyl–protected amino acid as the acyl donor and an amino acid amide as the nucleophile, in the presence of alcalase-CLEA with 2,2,2-trifluoroethanol as additive, the dipeptide carboxybenzyl-l-Ala-l-Leu-NH_2_ was obtained with a 13% yield [64]. The yield obtained was further improved to 87% when *C. antarctica* lipase B was added to the solution, probably because the esterification equilibrium as mediated by *C. antarctica* lipase B was shifted to the ester side when the ester was consumed in the alcalase-CLEA mediated coupling reaction [64].

### 4.7. Substrate Mimetics

**Figure 3:**
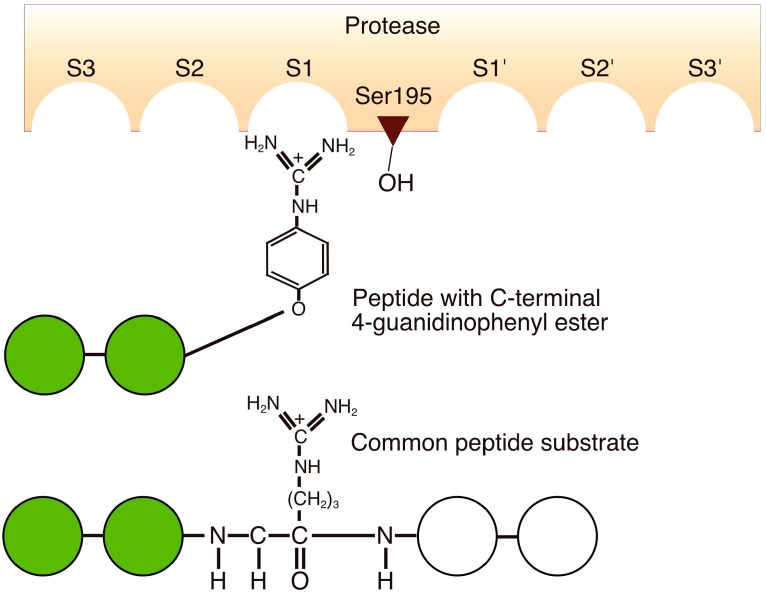
Schematic comparison of the binding of a peptide-4-guanidinophenyl ester and a common peptide substrate to the active site of trypsin based on the ideas of the conventional binding model of proteases.

To prevent protease-mediated hydrolysis, a substrate mimetic strategy was developed using the protease-recognized amino-acid side chain that was incorporated into an ester group. This enabled the identification of amino acids and peptide sequences that are not usually recognized by the enzyme [12,65]. The N-protected amino acid guanidine phenyl ester, which is a mimic of the arginine side chain recognized by trypsin, was the first example of substrate mimetics used for irreversible peptide bond formation (Figure 3) [65].

The guanidine phenyl ester group is widely used as part of the substrate mimetic approach. However, it has been found to exhibit some drawbacks because of which it is sensitive to spontaneous chemical hydrolysis and expensive to synthesize for large-scale applications [66]. Recently, benzyl and the dimethylaminophenyl esters were shown to be able to replace the benzyl group in the papain-catalyzed syntheses of dipeptide [66]. Benzyl and dimethylaminophenyl esters, which were obtained from the computational docking analysis among the set of esters, exhibit sufficient specific interactions with papain, although they do not possess an arginine-like activating ester recognized by papain. Papain-catalyzed dipeptide formation was performed using Phe-NH_2_ and *N*-carboxybenzyl-Gly with the activating ester of benzyl and dimethylaminophenyl. This resulted in 98.6% and 97.5% yields, respectively.

**Figure 4:**
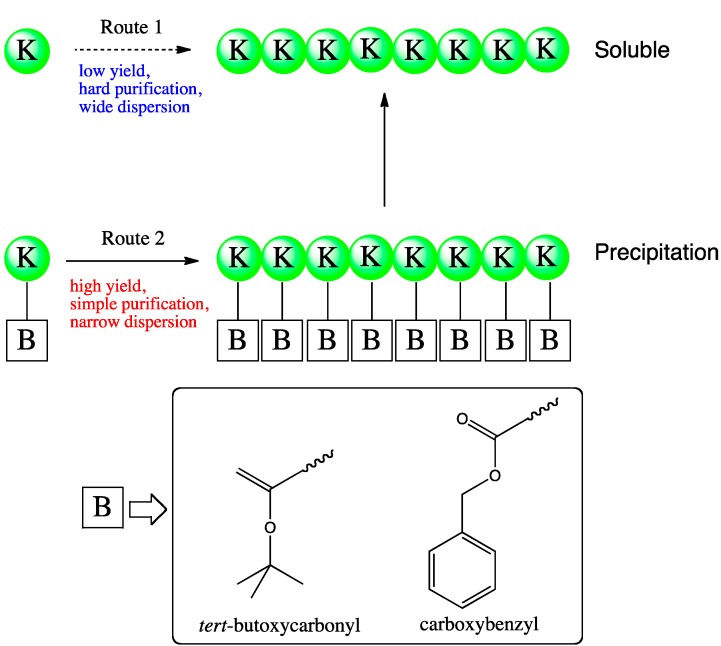
Papain-catalyzed synthesis of oligo(l-Lys) via direct oligomerization of l-lysine methyl ester (Route 1) or using the N_ε_-protected monomer l-lysine[B] methyl ester (Route 2). For the latter, B groups studied include *tert*-butoxycarbonyl and benzyloxycarbonyl.

Oligo(l-lysine) was synthesized with papain by using the N_ε_-protecting groups, viz., the *tert*-butoxycarbonyl and carboxybenzyl groups [37]. These protecting groups improved the hydrophobicity of the product, which led to facile purification and to a greater DP owing to the precipitation of products (Figure 4). Increasing the enzyme concentration to 1.89 mg/mL for 2 h reactions resulted in a 91% yield.

## 5. Various Applications using Chemoenzymatic Peptide Synthesis

### 5.1. Metal-Chelating Agents

**Figure 5:**
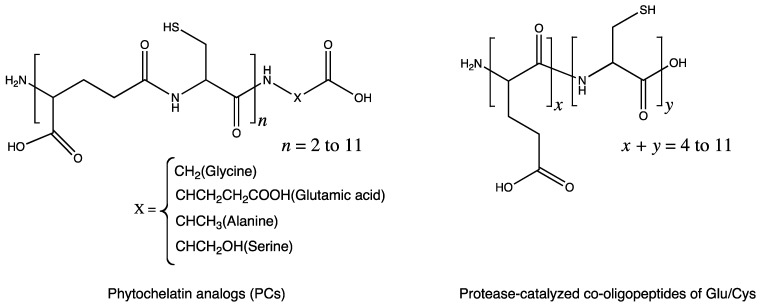
γ-Link between glutamate and cysteine residues in phytochelatin and α-link betweenglutamate and cysteine in co-oligopeptides synthesized by papain-catalyzed oligomerization.

Some peptides synthesized by chemoenzymatic approach were reported to show metal chelating abilities. Phytochelatin–like peptides were synthesized by employing papain-catalyzed oligomerization [18]. Phytochelatins consist of alternating γ-glutamic acid and cysteine residues, which are responsible for binding to heavy metals for cellular metal detoxification (Figure 5). Cysteine-rich peptides comprising l-Glu and l-Cys as potential phytochelatin mimics were prepared, which resulted in oligo(l-Glu-*co*-47%l-Cys) formation, by using an l-diethyl-Glu:l-ethyl-Cys feed ratio of 4:6 and a DP_avg_ of 9. The ability of the synthesized peptide to bind to the heavy divalent metals was about 50% that of the peptide with the perfect sequence.

Poly-l-cysteine was synthesized by α-chymotrypsin chemoenzymatically in a frozen aqueous solution serving as a metal chelating agent, which is still being tested for the removal of toxic metals from wastewater and polluted soils [25].

### 5.2. Antibiotics

**Figure 6:**
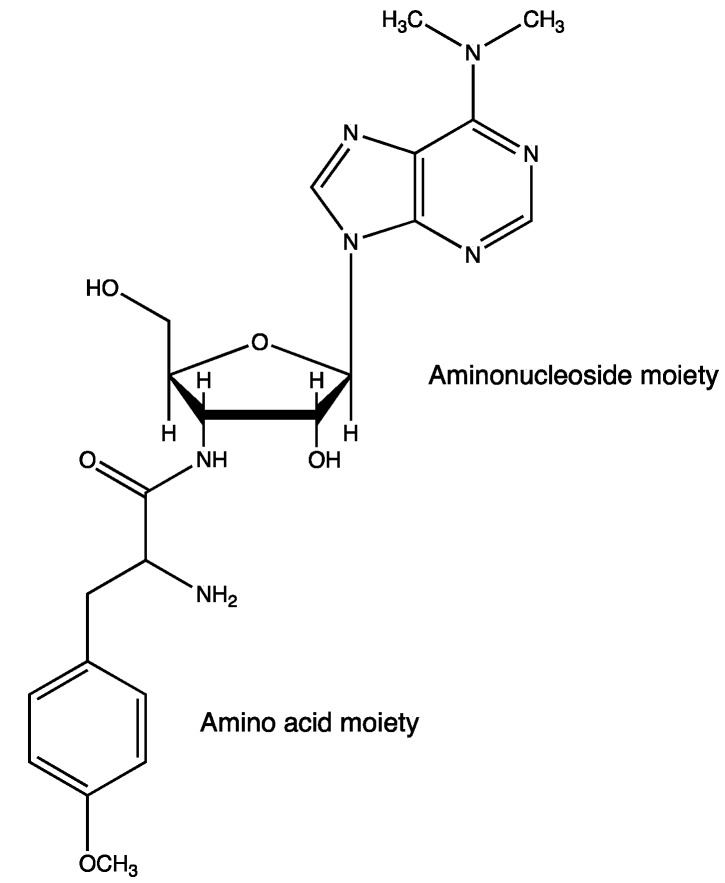
Structure of puromycin.

Puromycin is an aminoacyl nucleoside antibiotic, which is categorized as a mimic of aminoacyl-tRNA. It acts as an inhibitor of protein synthesis (Figure 6) [67,68].

Puromycin and its analogues are targets for the development of new antibiotics or chemical probes for biochemical studies. A new S9 family aminopeptidase derived from the actinobacterial thermophile *Acidothermus cellulolyticus* was cloned and engineered into a transaminopeptidase by site-directed mutagenesis of the catalytic Ser into Cys, which prevents the re-hydrolysis of the produced peptide [69]. The engineered biocatalyst, aminolysin-A, can catalyze the synthesis of diverse puromycin analogues composed of distinct amino acid residues. The engineered product exhibits antimicrobial activity similar to that of natural puromycin.

Cyclotides are a unique class of head-to-tail, cyclic cysteine-rich microproteins that have a length of up to 37 amino acids and exhibit a wide range of biological properties, ranging from anti-HIV to insecticidal activities [70]. MCoTI-II is the founding member of a small cyclotide family that possesses potent trypsin-inhibitory activity. Trypsin has a binding site in the MCoTI peptides at the active loop, Lys10-Ile11, and this characteristic was reversibly utilized to perform trypsin-catalyzed ligation between Lys10 and Ile11 of MCoTI peptides [70]. This ligation resulted in the formation of a peptide bond between Lys10-Ile11 with a 92% yield, in the phosphate buffer (pH 7.0). It exhibited inhibitory activities against trypsin, with *K*_i_ values similar to those reported for the corresponding natural products.

**Figure 7:**
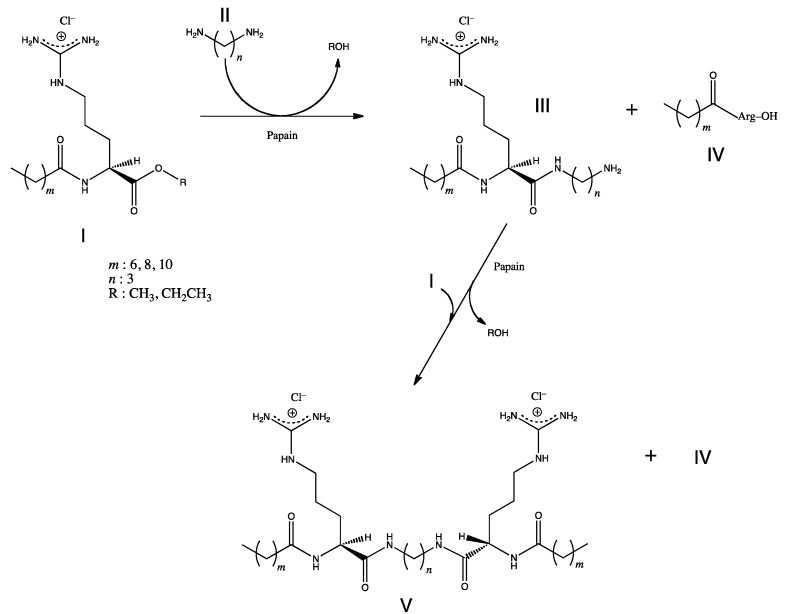
Scheme of the enzymatic synthesis of arginine-based gemini cationic surfactants: N^α^-acyl-l-Arginine alkyl ester monohydrochloride (acyl-donor) **I**, α,ω-diaminoalkane **II**, N^α^-acyl-l-arginine(ω-aminoalkyl)amide monohydrochloride **III**, N^α^-acyl-l-Arginine monohydrochloride **IV**, bis(N^α^-acyl-l-arginine) α,ω-polymetilenediamide dihydrochloride **V**.

### 5.3. Surfactants

Gemini or dimeric surfactants have attracted much interest due to the several unexpected properties superior to those of conventional monomeric surfactants [71]. They have excellent solubilizing, wetting, foaming, and antimicrobial properties. In addition, they have great potential for synergism, improving efficacy and effectiveness of other surface-active compounds, which make them considered as surfactants of the next generation. Arginine-based gemini cationic surfactants, which consist of two single N^α^-acyl-arginine structures connected through the α-carboxylic groups of the arginine residues by a α-ω-diaminoalkane spacer chain, were synthesized by papain-catalyzed reaction using N^α^-acyl-arginine alkyl ester derivatives as the starting building blocks (Figure 7) [72].

### 5.4. Potential Use in Tissue Engineering

The arginine-glycine-aspartate (RGD) motif plays a key role in mediating integrin-matrix interaction. RGD and RGD-containing peptides can serve as competitive, reversible inhibitors for the binding of adhesive proteins and have been used to study the adhesive interaction between cells and suppress tumor metastasis and platelet aggregation [31,73,74]. Trypsin-catalyzed synthesis of tetra peptide Bz-Arg-Gly-Asp-Ser-NH_2_ was conducted as a precursor of cellular adhesion motif of RGDS [73]. The tripeptide Gly-Asp-Ser-NH_2_ was synthesized by a chemical method and the linkage of the fourth amino acid (Bz-Arg-OEt) to GDS-NH_2_ was catalyzed by trypsin in ethanol/Tris-HCl buffer system (97/3, v/v) for 14 h with a yield of 68%.

**Figure 8:**
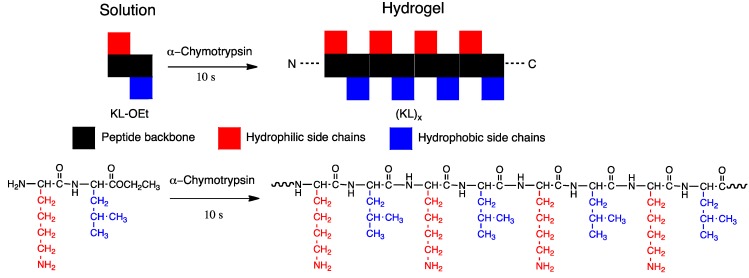
α-chymotrypsin-triggered hydrogelation via self-assembly of alternate oligomer of KL-OEt.

The enzyme α-chymotrypsin catalyzes the oligomerization of the “peptide lego”, as reported previously (Figure 8) [26]. Lysine-leucine ethyl ester was rapidly converted (less than 10 s) into oligopeptides by α-chymotrypsin, resulting in mixed chain oligomers that self-assembled into β-sheets at alkaline pH. Block and random oligo(l-lysine-*co*-l-alanine) were synthesized using catalysis of papain for short 30 min’ reaction and over 40% yield [36]. The crystalline structure observed by optical microscopy differed between random and block co-oligopeptides. At pH 3.0, diblock co-oligopeptides formed cubic or hexagonal crystals with a hollow structure; in contrast, at pH 7.4, they had an irregular shape [36]. These various structures open up several possibilities for the generation of materials that could be useful in tissue engineering.

### 5.5. Analgesics

Endomorphin-1 (Tyr-Pro-Trp-Phe-NH_2_, EM-1) has emerged as analgesics of comparable potency to morphine because morphine has a number of side effects such as physical dependence. EM-1 was synthesized by a combination of enzymatic and chemical methods [75]. Boc-Trp-Phe-NH_2_ was synthesized by the solvent-stable protease WQ9-2 in a 20% methanol medium with a yield of 97.1% and then Boc-Trp-Phe-NH_2_ was used to synthesize Boc-Tyr-Pro-Trp-Phe-NH_2_ by another organic solvent-tolerant protease, PT121 with chemically synthesized Boc-Tyr-Pro-OH in an organic–aqueous biphasic system with a yield of 84.5%.

### 5.6. Adhesive Peptides

**Figure 9:**
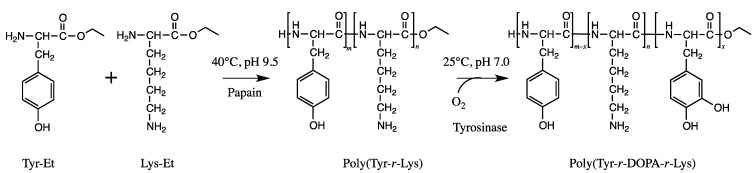
Two step chemoenzymatic synthesis of adhesive peptides containing DOPA and l-lysine.

The blue mussel (*Mytilus edulis*) foot protein 5 (Mefp-5) is an adhesive protein that can be found on the surface of the mussel adhesive plaque and is mainly composed of glycine, l-lysine, and 3,4-dihydroxy-l-phenylalanine (DOPA) [22]. Adhesive peptides similar to Mefp-5 were synthesized via two enzymatic reactions, viz. the papain-catalyzed polymerization of l-tyrosine and l-lysine and the tyrosinase-catalyzed conversion of tyrosine to DOPA (Figure 9) [22]. The synthesized peptides consisting of 50 mol% Tyr, 25 mol% DOPA, and 25 mol% Lys showed an adhesive strength higher than that of Super Glue. The authors proposed an adhesion mechanism in which deprotonated DOPA interacts with the surface materials and works as an adhesive molecule, while the primary amine group of lysine induces molecular networks under deprotonated conditions.

## 6. Conclusions and Future Perspectives

In this review, we have showcased the recent advances in chemoenzymatic peptide synthesis and have discussed the corresponding reaction mechanisms, reaction conditions, and versatile applications of the synthesized peptides. Chemoenzymatic peptide synthesis could serve as an alternative to conventional chemical peptide synthesis, because of its higher yields and lower use of toxic chemicals. Certain drawbacks such as the difficulty in controlling the sequences and a difficulty in synthesizing longer chains need to be addressed. The engineered proteases, substrate mimetics, and various reaction media, need to be combined to overcome the present issues. Enzymes cannot be universal for any substrate because of their substrate specificity, and therefore each reaction condition need to be optimized for various peptide synthesis schemes. Furthermore, the selection and engineering of the enzymes and substrates is likely to increase the applications of the chemoenzymatic peptide synthesis.
